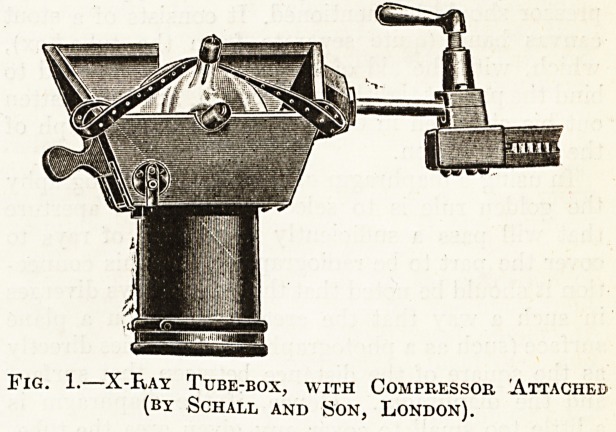# Fitting up the X-Ray Department

**Published:** 1912-10-26

**Authors:** Alfred C. Norman

**Affiliations:** House Surgeon at Durham County Eye Infirmary.


### ELECTRICITY IN MODERN MEDICINE.*

### XX.—Fitting up the X-Ray Department.

By ALFRED C. NORMAN, M.D. Edin., House Surgeon at Durham County Eye Infirmary.

In describing the fitting up of the interrupter the following paragraph should supplement the last section:—

The reservoir of the interrupter should be opened and the exact quantity of mercury indicated by the makers inserted, together with some paraffin or alcohol if one of these di-electrics is to be used (see page 123, The Hospital for May 4). If coal-gas is to be used as the di-electric, a metal gas-pipe must be brought along the wall to within a foot of the interrupter and there be made to terminate in a nozzle with a tap. The nozzle should be connected with the inlet pipe of the interrupter by a short length of rubber tubing and all the taps should be opened until gas flows freely from the outlet pipe of the interrupter; then the tap of the latter should be closed. When the interrupter is in use the inlet taps should be kept open and the outlet tap closed, but the latter should occasionally be opened for a few seconds in order to fill the reservoir with fresh gas.

---

It was intended to give in this week's issue a descriptive diagram of the writer's overhead frame, but it has been unavoidably held over until the next section. There are, however, some other accessories to be mentioned in connection with the equipment of the *x*-ray room before we go on to the consideration of radiography and fluoroscopy.

### The X-Ray Couch.

The couch should be as plain and as rigid as possible, and should contain few movable parts. The writer prefers a solid wood structure, similar to the old-fashioned operating tables, and it is an advantage if provision be made for raising and lowering the head end.

Some workers prefer to take radiographs with the *x*-ray tube underneath the couch and the photographic plate above the patient. If this method be adopted a special couch will be required with rails on the under side to support the tube-box and with a canvas top to allow of the *x*-rays passing through without obstruction. The disadvantages of this method are that there is greater risk of coming into contact with high-tension wires, since they are placed near the floor, and that the tube-box is not so easily accessible when it is necessary to adjust the diaphragm of the box or to regulate the vacuum of the tube.

### Tube-box, Diaphragms, and Stand.

*The tube-box* serves two purposes, which are nowadays considered to be of the utmost importance—it protects the operator from those *x*-rays which are given off at the side of the tube, and, with the aid of diaphragms, it prevents all but the central rays from reaching the patient and the photographic plate. Tube-boxes are made of wood and are lined with "lead-rubber," which is a non-conductor of electricity and is opaque to *x*-rays. Provision is made for accurately centring the anticathode opposite the aperture in the bottom of the box through which the rays are permitted to pass, and there is also some device for fixing various metal diaphragms over this aperture. For radiographic purposes it is sufficient if the box enclose the luminous portion of the tube, because this is the only part giving out *x*-rays; but for screen work it is an advantage to have a box large enough to completely enclose the tube, so that there shall be complete darkness in the room.

Fig. 1 illustrates an excellent type of tube-box which can be used in conjunction with diaphragms, therapeutic funnels, or a compressor cylinder. The *x*-ray tube is held in place by means of an elastic band, and the supports for the tube are so shaped that the latter is always accurately centred to the aperture in the diaphragm.

*Diaphragms* are used to prevent any rays but the *x*-rays emitted by the focus point of the anticathode

Fig. 1.—X-Ray Tube-box, with Compressor Attached (by Schall and Son, London).

from reaching the photographic plate. A considerable quantity of rays, known as secondary rays, are given off from the glass walls of the tube, and these, if allowed to reach the photographic plate, would produce a blurred radiograph, just as the light from a number of candles would produce an indistinct shadow, hence our reason for using diaphragms.

The simplest form of diaphragm consists of a sheet of metal, opaque to *x*-rays, but having a circular aperture through which the central rays may pass. A set of these diaphragms should contain apertures varying from ½ inch to 3 inches in diameter. A very useful form of variable diaphragm is a modification of the well-known iris diaphragm used

* Previous articles appeared on Nov. 11, 25, Dec. 9, 30, Jan. 13, 27, Feb. 17, March 9, 30, April 20, May 4, 25, June 8, July 6, Aug. 3. 17, 31, Sept. 28, and Oct. 12.

in photography; it is, of course, constructed on a much larger scale for *x*-ray work, and the segments are made of *x*-ray-proof metal.

*Cylinder diaphragms—i.e.*, diaphragms in the form of a tube—have been found to be more efficient than flat ones in cutting off secondary rays, and are now frequently used for radiography.

Lead-glass cylinders or funnels opaque to *x*-rays are largely used in giving therapeutic applications. A cylinder is selected with an aperture of the exact size of the area to be treated, and this is applied directly to the patient's body, thus limiting the action of the rays to the desired spot.

*Compressor diaphragms* are used in radiographing deep-seated organs, such as the kidney or hip-joint, in order to flatten out the intervening tissues, and thus give the *x*-rays less distance to travel through the body. The compressor takes the form of a heavy metal cylinder fitted into the aperture of the tube-box, and arranged in such a way that it can be applied very firmly to the patient's body, either by means of a special system of levers or by simply racking down the horizontal arm of the tube-stand. Fig. 1 shows the compressor *in situ*.

A compressor has three advantages: it keeps the part still; it renders the tissues anæmic and so allows the rays to pass through them more freely, for blood is somewhat opaque to *x*-rays; and it reduces the thickness of the tissues, thus lessening the distance the *x*-rays have to travel through them.*

Before leaving the subject another type of compressor should be mentioned. It consists of a stout canvas band (quite separate from the tube-box), which, with the aid of a special clamp, is used to bind the patient tightly to the couch, and thus flatten out his abdomen in order to secure a radiograph of the kidney region.

In using a diaphragm of any kind for radiography the golden rule is to select the smallest aperture that will pass a sufficiently large cone of rays to cover the part to be radiographed. In this connection it should be noted that the cone of rays diverges in such a way that the area it covers on a plane surface (such as a photographic plate) varies directly as the square of the distance between that surface and the diaphragm. Hence, if the diaphragm is a little too small to cover any given area the tube-box should be moved a shade farther away from the patient and the exposure be slightly prolonged.

*The tube-stand* is simply a support for the tube-box. It must be provided with an arrangement for raising and lowering the latter and with a universal joint so that the box may be rotated in any plane. The details must largely depend upon the type of work to be done and upon the amount of money at the disposal of the purchaser. An elaborate stand, such as the Beclere, is a thing of beauty and a joy for ever; but, on the other hand, excellent work can be done with the simple type supplied by most makers for about £7, complete with tube-box.

### The Fluorescent Screen.

The fluorescent screen consists of a sheet of cardboard coated with a thin layer of barium platino-cyanide. The screen is mounted on a wood frame and should be covered by a sheet of lead-glass in order to protect the user's face and eyes from the *x*-rays. The handles of the frame should be guarded by metal shields in order to shut off the *x*-rays from the hands of the operator. When the *x*-rays, which are, of course, invisible, fall upon the screen their presence is made known by an intense fluorescence set up in the barium platino-cyanide, and it is this fluorescence which allows us to examine directly the shadows of various deep-seated structures of the body that happen to be sufficiently opaque to *x*-rays.

### Radiometers.

We shall see in the section on radiography that the penetrating power of the *x*-rays emitted by a given tube has a very important bearing on the quality of the radiograph. In the past it has been the custom to express the penetrating power of the tube in terms of its equivalent spark-gap, but for many reasons this method gives only an approximate indication, hence the need for some kind of radiometer that will give a direct reading of the penetrating power of the tube.

Wehnelt's radiometer is a satisfactory instrument for the purpose. It consists of a thin strip of silver of uniform thickness, placed side by side with a wedge-shaped strip of aluminium of increasing thickness. The silver lets through almost the same amount of *x*-rays whether the tube be hard or soft, whereas the aluminium obstructs the rays in direct proportion to its thickness. If the instrument be held in front of a fluorescent screen while the tube is working the *x*-rays passing through only a certain section of the aluminium wedge will have the same intensity as those passing through the silver, and the thicker the section the higher we know the penetrating power of the tube to be. The different sections of the wedge are graduated in units from 1 to 15, and the reading is made directly by noting which section of the aluminium wedge allows the same intensity of fluorescence on the screen as the silver strip. The units are a reliable standard of comparison, recognised everywhere, and with their aid uniform radiography becomes a comparatively simple matter.

The small Wehnelt radiometer costs only a few shillings, and it has the advantage that it can be placed on a corner of the photographic plate when a radiograph is being taken, thus leaving a permanent record of the penetration of the tube used in every instance.

It should be noted that this instrument only measures the *penetrating power* of the *x*-rays, not their *quantity*.

*(To be continued.)*

---

* It is now recognised that fresh *x*-rays, known as secondary rays, are generated whenever the direct *x*-rays strike a solid substance, such as metal, glass, or the various tissues of the human body, and that these secondary rays tend to spoil the sharpness of the radiograph. Hence the advantage of flattening out the tissues by compression, for the proportion of secondary rays varies with the thickness of tissue through which the direct rays pass.

## Figures and Tables

**Fig. 1. f1:**